# A New Human Cancer Cell Proliferation Inhibition Sesquiterpene, Dryofraterpene A, from Medicinal Plant *Dryopteris fragrans* (L.) Schott

**DOI:** 10.3390/molecules22010180

**Published:** 2017-01-21

**Authors:** Zheng-Chang Zhong, Dan-Dan Zhao, Zhen-Dong Liu, Shuai Jiang, Yan-Long Zhang

**Affiliations:** 1 Food Science Department of XiZang Agriculture and Animal Husbandry College, Xizang 860000, China; zxzzc@163.com (Z.-C.Z.); liu304418091@126.com (Z.-D.L.); 2 Key Laboratory of Molecular Biology of Heilongjiang Province, College of Life Science, Heilongjiang University, Harbin 150080, China; zhaodandan@hlju.edu.cn (D.-D.Z.); jiangshuaixbxb@foxmail.com (S.J.)

**Keywords:** *Dryopteris fragrans* (L.) Schott, dryofraterpene A, cancer cell, proliferation inhibition

## Abstract

The global burden of cancer continues to increase largely with the aging and growth of the world population. The purpose of the present work was to find new anticancer molecules from a natural source. We utilized chromatographic methods to isolate compounds from medicinal plant *Dryopteris fragrans* (L.) Schott. The structure of the new compounds was determined by spectroscopic and spectrometric data (1D NMR, 2D NMR, and EMI-MS). Their anti-proliferation effects against five human cancer cell lines including A549, MCF7, HepG2, HeLa, and PC-3 were evaluated by CCK-8 andlactate dehydrogenase (LDH) assay. A new sesquiterpene, (7*S*, 10*S*)-2,3-dihydroxy-calamenene-15-carboxylic acid methyl ester (**1**), and two known compounds (**2** and **3**) were isolated. The new sesquiterpene was named dryofraterpene A and significantly inhibited cancer cell proliferation without any obvious necrosis below a 10 μM concentration. In conclusion, a novel anticancer sesquiterpene together with two known compounds was isolated, which might be a promising lead compound for the treatment of cancer.

## 1. Introduction

Cancer is a major public health problem in a great many parts of the world. In the United States alone, a total of 577,190, 580,350, 585,720, 589,430, and 595,690 deaths from cancer were respectively predicted to occur in 2012–2016 [1,2,3,4,5], and these numbers have been increasing year by year. Great therapy attention has been paid to the development of novel anticancer molecules from natural sources. However, the available chemotherapeutics is often limited due to undesirable drug resistance and side effects [6,7]. It is urgent that new targets for the treatment of cancer are identified. 

**Figure 1:**
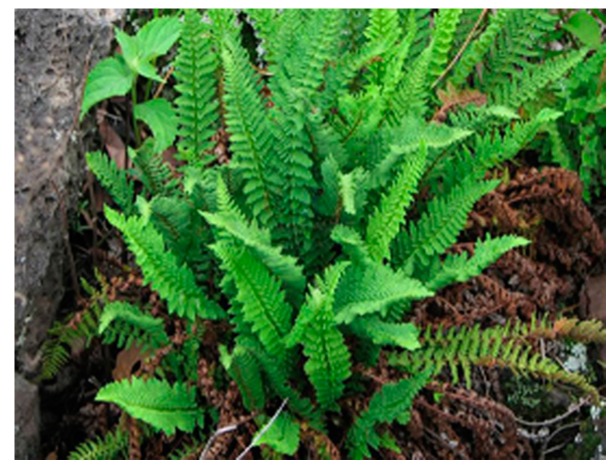
*Dryopteris fragrans*.

*Dryopteris fragrans* (L.) Schott (Chinese name: Xiang-Lin-Mao-Jue) (Figure 1), a deciduous perennial herb from the family Dryopteridaceae, is widely distributed in Asia-temperate, Europe, and North America [8]. In the north of China, it has drawn wide attention due to its folk effect on various dermatosis and rheumatoid arthritis [9]. Many constituents of *D. fragrans* have exhibited various biological activities, such as anticancer [10], anti-inflammatory [11], antibacterial [12], antifungal [13], and antioxidant activities [14].

**Figure 2:**
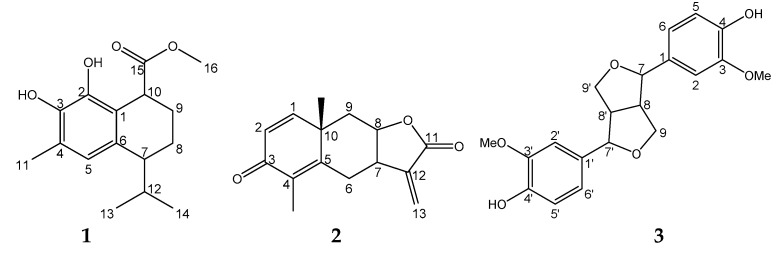
Structures of **1**–**3** isolated from *D. fragrans.*

Therefore, with the intent of discovering new compounds with potential antitumor properties, we performed two fractionations of the EtOAc extract and obtained a new sesquiterpene (**1**) together with two known compounds (**2** and **3**) (Figure 2). We are strongly interested in the biological activity of the new sesquiterpene. Human cancer cell proliferation inhibition activity was tested on A549, MCF7, HepG2, HeLa, and PC-3 cells, which are the in vitro models of lung, breast, liver, cervical, and prostate cancer that are the leading cause of cancer death in more or less developed countries [15]. The novel sesquiterpene showed the inhibitory effects of cancer cell growth.

## 2. Results and Discussion 

### 2.1. Identification of Isolated Compounds

Compound **1** was colorless crystals, m.p. 115 °C, [α${]}_{D}^{25}$ −37.9 (c 0.0026, CDCl_3_). The molecular formula was assigned as C_16_H_22_O_4_ on the basis of the [M + H]^+^ peak at *m*/*z* 279.1590 (Calcd for C_16_H_23_O_4_, 279.1596) in its high resolution electrospray ionization mass spectroscopy (HR-ESI-MS), and this could be supported by evidence from ^13^C-NMR combined with the distortionless enhancement by polarization transfer (DEPT) spectrum (Table 1). The infrared radiation (IR) spectrum of compound **1** showed a presence of the hydroxyl group (3431 cm^−1^), an aromatic ring (1629 cm^−1^), and an ester carbonyl group (1721 cm^−1^).

The ^13^C-NMR and DEPT spectrum showed 16 carbon signals (four methyl, two methylene and four methine groups, and six quaternary carbon atoms). All of these signals indicated compound **1** was cadinene sesquiterpene. By detailed analyses of the ^1^H-NMR (Table 1), only one aromatic proton could be observed (δ_H_ 6.60, 1H, s), which indicated the aromatic ring was quinque-substituted. While two methyl groups belonged to the isopropyl group (δ_H_ 0.99, d, *J* = 6.8 Hz, 3H; 0.78, d, *J* = 6.8 Hz, 3H), one methyl group attached to benzene ring (δ_H_ 2.21, 3H, s), one oxygenated methyl (δ_H_ 3.73, 3H, s), two methylene groups (δ_H_ 1.74–1.81, m, 2H; 1.88–1.97, m, 2H), and methine groups (δ_H_ 3.93, t, *J* = 5.7 Hz, 1H; 2.50, q, *J* = 4.5 Hz, 1H; 2.0–2.12, m, 1H) were observed.

**Figure 3:**
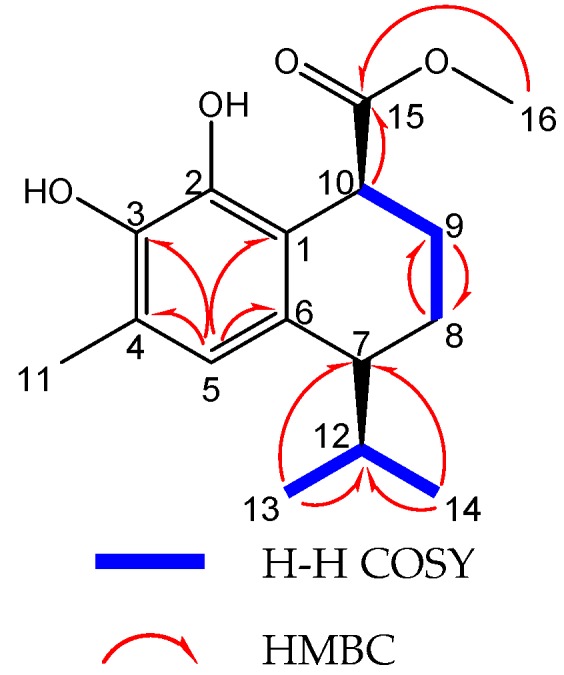
^1^H-^1^H correlated spectroscopy (COSY) and detected heteronuclear multiple bond correlation HMBC of **1**.

In the ^1^H detected heteronuclear multiple bond correlation (HMBC) spectrum of compound **1** (Figure 3), long-range correlation from H-11 (δ_H_ 2.21) to C-3, C-4, and C-5 (δ_C_ 141.5, 118.8, 123.3) and the correlation from H-5 (δ_H_ 6.60) to C-4 and C-11 (δ_C_ 118.8, 15.6) indicated that the isolated methyl group was linked to C-4. Long-range correlations from H-9 (δ_H_ 1.88–1.97) and H-10 (δ_H_ 3.93) to C-15 (δ_C_ 177.2) confirmed that the ester was located at C-10. In addition, the H-7 (δ_H_ 2.50) exhibited three-bond correlations with C-13 and C-14 (δ_C_ 21.9, 19.0), suggesting that the isopropyl group was fused to C-7 (Table 1).

The 2D-nuclear overhauser effect spectroscopy (NOESY) spectrum established the relative configuration of the stereocenters for compound **1**. A correlation between Me-16 and Me-14 confirmed a *cis*-calamenene, according to reported literature [16,17]. Based on the above spectroscopic analysis, the structure of compound **1** was determined to be (7*S*, 10*S*)-2,3-dihydroxy-calamenene-15-carboxylic acid methyl ester and was named as dryofraterpene A.

The structures of known compounds were identified as yomogin (**2**) [18] and pinoresinol (**3**) [19] by spectroscopic (^1^H-NMR, ^13^C-NMR and DEPT) measurements and by comparison with published data.

### 2.2. Effects of Compounds on Cancer Cell Proliferation

By the CCK-8 assay, dryofraterpene A (**1**) was evaluated for cancer cell proliferation inhibition activities in vitro against A549 (lung cancer), MCF7 (breast cancer), HepG2 (liver cancer), HeLa (cervical cancer), and PC-3 (prostate cancer) human cell lines, using taxol as a positive control. As summarized in Table 2, dryofraterpene A (**1**) significantly inhibited the growth of all the five cell lines. At the same time, we observed a decrease in the total cell number and an increase in floating cells, with cell shrinkage and cytoplasm vacuolization in dryofraterpene A-treated cancer cells by the inverted phase-contrast microscope (data not shown).

The LDH assay detects the amount of LDH released by cells with damaged membranes as indicator of necrosis. Forty-eight-hour treatment with dryofraterpene A (**1**) did not affect the concentration of LDH in the supernatant of culture medium of five cancer cell lines (99.9% ± 8.7%, 103.2% ± 7.0%, 98.2% ± 6.4%, 100.5% ± 4.3%, and 101.6% ± 7.4% at 10 μM, respectively, *p* > 0.05). This suggests an anti-proliferative effect of dryofraterpene A (**1**) on cancer cells without any obvious necrosis, perhaps with inducing apoptosis, below the dose of at least 10 μM, which might be used for treatment in future experiments [20].

## 3. Materials and Methods 

### 3.1. General Procedures

Melting points were obtained on an Yanaco micro melting point apparatus (Yanaco, Beijing, China). Optical rotations were measured on a JASCO DIP-370 digital polarimeter (JASCO, Tokyo, Japan). IR spectra were obtained on a Bruker Tensor 27 spectrometer (Bruker Optics, Inc., Billerica, MA, USA) with KBr pellets. Mass spectrometry (including HR-ESI-MS) was carried out on VG Autospec-3000 mass spectrometers (VG, Manchester, England). 1D and 2D NMR spectra was performed on Bruker AM-400 (Bruker, Fällanden, Switzerland) spectrometers with tetramethyl silane (TMS) as an internal standard. Column chromatography was performed on silica gel (SiO_2_: 200–300 and 100–200 mesh, Qingdao Marine Chemical Ltd., Qingdao, China) and MPLC gel (75–150 μm; Mitsubishi Chemical Corporation, Tokyo, Japan). Semi-preparative HPLC was performed on an Agilent 1100 liquid chromatography (Agilent Technology Inc., Urdorf, Switzerland). Fractions were monitored using thin-layer chromatography (TLC), and spots were visualized by heating silica gel plates (G254, Qingdao Marine Chemical Ltd., Qingdao, China) immersed with 10% H_2_SO_4_ in ethanol.

### 3.2. Plant Material

*Dryopteris fragrans* (L.) Schott was collected in Wu-Da-Lian-Chi, Heilongjiang Province, China, in August 2009, and identified by Prof. Zhen-Yue Wang (Heilongjiang University of Chinese Medicine). The voucher specimen (Registration number: XLMJ-20110812) of this plant was deposited in the Herbarium of Heilongjiang University of Chinese Medicine, Harbin, China.

### 3.3. Extraction and Isolation

Air-dried, powdered whole plants of *D. fragrans* (3 kg) were extracted three times with 95% ethanol at room temperature. After removal of the solvent by evaporation, the residue (240 g) was suspended in H_2_O and partitioned with EtOAc. The EtOAc fraction (135 g) was subjected to silica gel column chromatography with a gradient elution system of petroleum ether–acetone (90:10–0:100, *v*/*v*) to obtain five fractions (FrI–FrV). FrII was fractionated bymedium pressure liquid chromatography (MPLC), eluting with MeOH–H_2_O (90:10–0:100, *v*/*v*), to provide five fractions (FrII1–FrII5). FrII1 was subjected to silica gel column chromatography, eluting with petroleum ether–acetone (90:10–0:100, *v*/*v*), to afford FrII11–FrII1-5. FrII1-1 was chromatographed over silica gel eluting with CHCl_3_–Me_2_CO (85:15, *v*/*v*) to produce crystals. The crystals were eluted with petroleum ether and detected by HPLC to obtain compound **2**. FrII2 was separated using a Sephadex LH-20 column chromatography with CHCl_3_ and MeOH mixture (1:1 *v*/*v*), yielding FrII2-1. FrII2-1 was purified by semi-preparative HPLC (MeOH/H_2_O, 65:35, eluting for 20 min with a flow rate of 30 mL/min) to afford compound **1**.

FrIII was decolorized with MPLC (MeOH/H_2_O, 65:35, *v*/*v*) and fractionated by octadecyl-silica (ODS) column (MeOH/H_2_O, 10:90–100:0, *v*/*v*) to provide five fractions (FrIII1–FrIII5). FrIII3 was isolated by Sephadex LH-20 (CHCl_3_/MeOH, 1:1, *v*/*v*) and silica gel column chromatography (CHCl_3_/MeOH, 200:1, *v*/*v*) to obtain FrIII3-1 and FrIII3-2. FrIII3-2 was purified by Sephadex LH-20 column chromatography (CHCl_3_/H_2_O, 80:20, *v*/*v*) to yield compound **3**.

### 3.4. Spectral Data

Dryofraterpene A (**1**): colorless crystals; IR (KBr)ν_max_ 3431, 2922, 2852, 1721, 1628, 1461 cm^−1^; ^1^H- and ^13^C-NMR data, see Table 1; ESI-MS: *m*/*z* 301 [M + Na]^+^; HR-ESI-MS: *m*/*z* 301.1590 [M + Na]^+^; Calcd for C_14_H_16_O_4_Na, 271.1133.

### 3.5. Cell Culture

Human A549, MCF7, HepG2, HeLa and PC-3 cells were obtained from Cell Library of Committee on Type Culture Collection of Chinese Academy of Sciences. Cultures were maintained in 95% air and 5% CO_2_ at 37 °C in Roswell Park Memorial Institute (RPMI) 1640 medium with 10% FBS, 2 mmol/L l-glutamine, 100 U/mL penicillin, and 100 U/mL streptomycin.

### 3.6. Cell Counting Kit-8 Assay

Cancer cell proliferation inhibition activity was measured using a CCK-8 assay [21]. Cell Counting Kit-8 (CCK-8), 2-(2-methoxy-4-nitrophenyl)-3-(4-nitrophenyl)-5-(2,4-disulfophenyl)- disulfophenyl)-2H-tetrazolium, was obtained from Dojindo Laboratories (Kumamoto, Japan). A stock solution of 10 mM dryofraterpene A (**1**) was prepared in sterilized dimethyl sulfoxide (DMSO) and further diluted to appropriate concentrations with a cell culture medium immediately before use. All five cells (2 × 10^3^ cells/mL in 96-well culture plates) were treated with various concentrations of dryofraterpene A (**1**) (0, 0.08, 0.4, 2, 10, and 50 μM) for 48 h. The medium (90 μL) was incubated with 10 μL of CCK-8 solution for 2 h at 37 °C. Absorbance was measured at 450 nm in a plate microreader (TECAN Infinite 200, Eastwin Life Science, Beijing, China). IC_50_ values, the concentration of the test compounds inhibiting 50% of the cell growth at 48 h, was calculated by Reed and Muench’s method [22]. Data were obtained from three independent assays. Taxol was used as positive control (0, 0.08, 0.4, 2, 10, and 50 μM for 48 h) [23,24].

### 3.7. LDH Assay

Leakage of LDH to the cell culture medium indicates cell membrane damage. LDH assay kit was purchased from Jiancheng Bioengineering Institute (Nanjing, China). After cells were exposed to dryofraterpene A (**1**) (0 and 10 μM) for 48 h, each culture medium was centrifuged at 250 *g* for 10 min. Supernatant was transferred to a 96-well culture plate to determine the amount of LDH according to the manual of the LDH assay kit. LDH activity is reported as a percentage relative to control level [25]. Absorbance of samples was measured at 450 nm. Data were obtained from three independent assays.

## 4. Conclusions 

In summary, dryofraterpene A, a new sesquiterpene, (7*S*, 10*S*)-2, 3-dihydroxy-calamenene-15-carboxylic acid methyl ester, was isolated from medicinal plant *D. fragrans*, and could significantly inhibit tumor cells proliferation including A549, MCF7, HepG2, HeLa, and PC-3 cancer cells. However, a defined mechanism should be further studied.

## Figures and Tables

**Table 1:** ^1^H (400 MHz) and ^13^C-NMR (100 MHz) data of compound **1** in CDCl_3_.

No.	δ_C_	δ_H_ (*J* in Hz)	No.	δ_C_	δ_H_ (*J* in Hz)
1	122.6 (C)		9	22.2 (CH_2_)	1.76–1.83 (1H, m)
2	131.8 (C)		10	39.7 (CH)	3.93 (1H, t, 5.6)
3	141.5 (C)		11	15.6 (CH_3_)	2.21 (3H, s)
4	118.8 (C)		12	32.7 (CH)	2.05 (1H, m)
5	123.3 (CH)	6.60 (1H, s)	13	21.9 (CH_3_)	0.99 (3H, d, 6.8)
6	141.3 (C)		14	19.0 (CH_3_)	0.78 (3H, d, 6.8)
7	41.8 (CH)	2.50 (1H, q, 4.6)	15	177.2 (C)	
8	20.7 (CH_2_)	1.89–1.98 (1H, m)	16	52.7 (CH_3_)	3.73 (3H, s)

**Table 2:** In vitro cytotoxicity of dryofraterpene A against five cancer cell lines *.

Compound	A549	MCF7	HepG2	HeLa	PC-3
dryofraterpene A	2.84 ± 0.79	1.58 ± 0.47	3.53 ± 0.87	1.65 ± 0.45	4.62 ± 0.94
Taxol **	0.05 ± 0.04	0.12 ± 0.07	0.36 ± 0.11	0.04 ± 0.02	0.21 ± 0.13

* Results are expressed as IC_50_ values in μM, which represent the mean ± standard error (SE) of three independent assays. ** Taxol was as positive control.
